# Biarticular energy transfer mechanisms of the gastrocnemii muscles are associated with managing body energy during hole negotiation gait

**DOI:** 10.1038/s41598-026-44470-z

**Published:** 2026-03-31

**Authors:** Christos Theodorakis, Sebastian Bohm, Maria-Elissavet Nikolaidou, Morteza Ghasemi, Falk Mersmann, Adamantios Arampatzis

**Affiliations:** 1 Department of Training and Movement Sciences, Humboldt-Universität zu Berlin, Philippstr. 13, Haus 11, 10115 Berlin, Germany; 2 Berlin School of Movement Science, Humboldt-Universität zu Berlin, Berlin, Germany; 3 Division of Sport Medicine and Biology of Exercise, Faculty of Physical Education and Sport Science, National and Kapodistrian University of Athens, Athens, Greece

**Keywords:** Challenging locomotion, Energy transfer potential, Muscle activation, Vector coding, Joint kinematics, Uneven terrain, Anatomy, Health care, Medical research, Physiology

## Abstract

In this study, the energy transfer potential between the ankle and knee joints via the biarticular gastrocnemius medialis and lateralis muscles was investigated, as well as its association with changes in total centre of mass (CoM) energy during hole negotiation - a common task in daily life locomotion - and level walking. Whole-body kinematics and activation patterns of the gastrocnemii and vasti muscles were measured during the preparation, hole, and recovery steps in 18 participants. During hole negotiation, we found a significant (*p* < 0.001) increase in the peak-to-peak range of total CoM energy, providing evidence for an increased challenge in managing CoM energy. We observed significantly (*p* < 0.001) increased potential for energy transfer between the ankle and knee joints via the biarticular gastrocnemii muscles, accompanied by the gastrocnemii and vasti muscles being active during the energy transfer phases. Finally, we found that the increase in energy transfer potential from the ankle to the knee joint was associated with a decrease in total CoM energy, and the increase in energy transfer potential from the knee to the ankle joint was associated with an increase in total CoM energy. Our findings demonstrate increased involvement of biarticular mechanisms in the management of total CoM energy during hole negotiation compared to level walking.

## Introduction

Biarticular mechanisms of the gastrocnemii muscles significantly affect performance during high-intensity activities such as jumping and accelerated running^1–3^. We recently observed a significant modulation of the energy transfer potential between the ankle and knee joints via the biarticular gastrocnemii muscles during trip- and drop-like perturbations^4^, as well as during sprinting^5^, highlighting the importance of biarticular mechanisms in these locomotor tasks. Key advantages of biarticular muscles are (a) their ability to enable adjacent monoarticular muscles to indirectly act at joints they do not span, and (b) their capacity to effectively act on either of the joints they span, as required^6^. Biarticular muscles, due to their ability to act simultaneously on two joints, can also reduce the cost of neural control of the musculoskeletal system^7^ and, through coactivation with adjacent monoarticular muscles, minimize the mechanical delay of the system in response to neural commands^2,8^. A reduction in the cost of neural control and mechanical delays to neural responses is crucial during fast movements or following locomotor perturbations^9–12^. In addition, biarticularity enables mechanical power and work to be transferred from the larger proximal leg muscles to the less voluminous distal leg muscles^8^. In particular, the biarticular gastrocnemii muscles can transfer power and energy to the ankle joint from the monoarticular vasti^1^, which are more voluminous than the plantarflexors^13,14^ and can therefore produce more mechanical power and work. Thus, independently of their own musculotendinous power and work production, the transferred mechanical power and work from the more proximal vasti muscles can enhance the mechanical power and work at the ankle joint^2,8,15^. Recent research has shown that although the contribution of the biarticular mechanisms of the gastrocnemii muscles to the mechanical power and work at the ankle joint is negligible during level walking at preferred and slow speeds^16^, this contribution increases significantly at fast and maximal walking speeds and during running, where the demand for mechanical power and work at the ankle joint is higher^16,17^.

Energy transfer between the ankle and knee joints via the biarticular gastrocnemii muscles can occur when the mechanical power of the gastrocnemii muscles at the two joints have opposite signs^15,17^. Conversely, when the mechanical powers of the gastrocnemii muscles at the two joints have the same sign, the gastrocnemii muscles are simultaneously absorbing or producing energy at both the ankle and knee joints^15,17^. As the gastrocnemii muscles generate plantarflexion moments at the ankle joint and flexion moments at the knee joint, during in-phase fluctuations at the two joints (i.e. synchronous knee extension and plantarflexion, or knee flexion and dorsiflexion), the mechanical power at the ankle and knee joints will have opposite signs, allowing energy to be transferred between the two joints^4^. During anti-phase fluctuations (i.e. synchronous knee flexion and plantarflexion, or knee extension and dorsiflexion), the mechanical power at the ankle and knee joints would have the same signs, indicating the possibility of simultaneous energy production or absorption at the two joints via the gastrocnemii muscles^4^. This approach demonstrates the feasibility of using ankle and knee joint angle trajectories to generate data on the involvement of biarticular mechanisms in locomotion^4,5^.

Real-world locomotion is primarily associated with movement on uneven terrain^18^ and is linked to external mechanical perturbations, increased gait instability, and an elevated risk of falls^19–21^. Studies of leg and joint mechanics have demonstrated the vital role of the ankle joint and triceps surae muscles in managing body energy and maintaining stability while moving on uneven terrain^22–25^. In everyday life, negotiating holes is a common movement task when walking on uneven terrain, and incorrect placement of a step can lead to falls^19,21^. When stepping down, the centre of mass (CoM) is lowered in the step before touchdown in the hole, resulting in greater fluctuations in the vertical CoM trajectory and, consequently, greater fluctuations in potential CoM energy during hole negotiation gait compared to level walking^22,24^. Increased electromyographic (EMG) activity in muscles of both the ipsilateral and contralateral legs has been reported during hole negotiation gait compared to level walking^24,26,27^, in order to manage the body energy required to negotiate the hole. The increased challenge of managing body energy during hole negotiation gait could require greater involvement of the biarticular mechanisms that transfer energy between the ankle and knee joints. To our knowledge, no information is available on how biarticular mechanisms may effectively support the neuromotor system in managing increased energy demands during hole negotiation. Understanding the role of biarticular mechanisms in controlling the increased demand during locomotion on uneven terrain could improve the design of prevention and rehabilitation treatments to enhance stability and prevent falls, as well as the design of powered prostheses and exoskeletons.

The current study investigated how biarticular mechanisms of the gastrocnemius medialis (GM) and gastrocnemius lateralis (GL) muscles are involved when negotiating a hole, and how these mechanisms are associated with the management of total CoM energy. We hypothesised (a) an increased potential for energy transfer between the ankle and knee joints through the biarticular gastrocnemii muscles during hole negotiation compared to level walking, and (b) a relationship between the joint energy transfer potential and fluctuations in total CoM energy, particularly a reduction in total CoM energy during the ankle-to-knee joint energy transfer phase, and an enhancement during the knee-to-ankle joint energy transfer phase.

## Methods

**Fig. 1:**
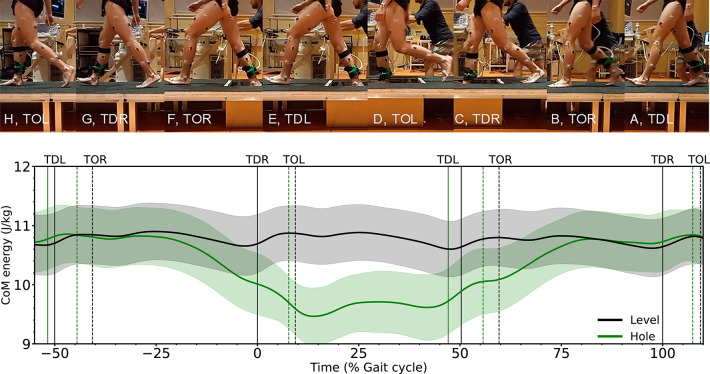
The images show the characteristic time points during the hole negotiation gait, touch-down of the left leg in the preparation step (**A**, TDL), take-off of the right leg in the preparation step (**B**, TOR), touch-down of the right leg in the hole (**C**, TDR), take-off of the left leg in the hole step (**D**, TOL), touch-down of the left leg in the recovery step (**E**, TDL), take-off of the right leg from the hole (**F**, TOR), touch-down of the right leg in the recovery step (**G**, TDR), and take-off of the left leg in the recovery step (**H**, TOL). The second row shows the total centre of mass (CoM) energy during level and hole negotiation walking. The curves and shaded areas represent mean ±standard deviation. The horizontal axis is normalized to the gait cycle before touch-down in the hole (negative percentages) and to the gait cycle after touch-down in the hole (positive percentages). The vertical solid lines show the touch-down of the left and the right leg and the vertical dashed lines show the take-off. Stance Pre: stance phase of the preparation step, Stance Hole: stance phase of the hole step, Stance Rec: stance phase of the recovery step.

In eighteen participants (5 female and 13 male, age 24.8 ±5.4 years, body mass 75.2 ±9.7 kg, height 176.5 ±7.8 cm) we investigated the hole negotiation gait by measuring whole body kinematics and bilateral EMG activity of the GM, GL, vastus medialis (VM) and vastus lateralis (VL) muscles. The study was approved by the Ethics Committee of the Humboldt-Universität zu Berlin (HU-KSBF-EK_2022_0032) and all participants gave written informed consent in accordance with the Declaration of Helsinki. Participants walked at their preferred walking speed (1.3 ±0.1 m/s) on an 18 m custom-built walkway and stepped with their right leg through a hole (15 cm deep, 70 cm long and 46 cm wide) located in the second half of the walkway (Fig. 1). Before starting the measurements, participants adjusted their starting position on the walkway to negotiate the hole with the right leg. Participants were secured against falling by a harness system. They performed three level walking and then five hole negotiation gait trials and the first level walking trial and the fifth hole negotiation gait trial were included in the analysis. We included the fifth negotiation trial in our study, to consider a visually guided, experience-based gait for negotiating holes. We analysed the stance phases of three consecutive steps (preparation: touch-down of the left leg before touch-down in the hole and until take-off, hole: touch-down of the right leg in the hole and until take-off, and recovery: touch-down of the left leg after touch-down in the hole and until take-off (Fig. 1)).

Whole-body kinematics were recorded using an infrared motion capture system (Vicon Nexus, version 2.12, Vicon Motion Systems, Oxford, UK) with 19 cameras operating at 250 Hz. The three-dimensional coordinates of 22 markers located on the second metatarsal, tuber calcaneus, lateral malleolus, lateral epicondyle, greater trochanter, acromion, elbow and wrist bilaterally, sacrum, 7th cervical vertebra and head (2 anterior and 2 posterior) were measured. A fourth-order low-pass Butterworth filter with zero phase shift and a cut-off frequency of 12 Hz was applied to the measured three-dimensional coordinates of the markers and, using the method presented by Maiwald et al.^28^, we determined the touch-down and take-off of the foot. In short, the maximum vertical acceleration of the second metatarsal marker was used to define the take-off of the foot. The maximum of the vertical acceleration of the calcaneus marker was used to define the touch-down in rear foot contact or the maximum of the second metatarsal marker in fore-foot contact. The lateral malleolus, lateral epicondyle and greater trochanter markers were used to calculate the knee joint angle and the second metatarsal, lateral malleolus and lateral epicondyle markers were used to calculate the ankle joint angle in relation to the upright stance position (180° for the knee and 0° for the ankle joint angles). Knee joint angles less than 180° indicate a flexed knee joint position, positive ankle joint angle values indicate plantarflexed and negative values indicate a dorsiflexed position. Step length was defined as the anteroposterior distance between the calcaneus markers of the two legs at the beginning of the double contact phase. Dorsiflexion of the ankle joint was defined as the difference between the ankle joint angle at touch-down and the maximum dorsiflexion during the stance phase. Ankle plantarflexion was defined as the difference between the ankle joint angle at take-off and maximum dorsiflexion. Finally, knee flexion was defined as the difference between the knee joint angle at touch-down and the first minimum of knee flexion during the stance phase. The total energy of the CoM was calculated as follows (Eq. 1):

1 $${E}_{CoM}=mg{h}_{CoM}+\frac{1}{2}m{{{v}}^{2}_{CoM}}$$

where m is the mass of the body (kg), g is the acceleration of gravity (m/s^2^), ${h}_{CoM}$ is the height of the CoM (m) and ${{v}}_{CoM}$ is the velocity vector of the body CoM (m/s). The masses of the segments and their positions within each segment to calculate the CoM were taken from Dempster^29^. For illustrative purposes, the time axis in the figures is normalised to the time of the gait cycles before and after touchdown in the hole.

Vector coding was used to determine the in-phase and anti-phase fluctuations of the ankle and knee joint angles during the stance phase of the preparation, hole and recovery steps, and the coupling angle (γ) of the ankle and knee joint angles was calculated using the equations reported by Needham et al.^30^. From the coupling angles, it was possible to identify the time during the stance phase when the biarticular gastrocnemii muscles can transfer energy from the ankle to the knee joint and vice versa, or absorb/produce energy at both joints simultaneously^4,5^. In short, a coupling angle of 0° ≤ γ < 90° indicates in-phase fluctuations between the ankle and knee joint angles and the possibility of knee-to-ankle joint energy transfer via the biarticular gastrocnemii muscles. A coupling angle of 90° ≤ γ < 180° indicates anti-phase fluctuations and the possibility of simultaneous energy production at both joints. A coupling angle of 180° ≤ γ < 270° again indicates in-phase fluctuations and the possibility of energy transfer from the ankle to the knee joint. Finally, a coupling angle of 270° ≤ γ < 360° indicates anti-phase fluctuations and the possibility of simultaneous energy absorption at both joints. The fraction of contact time during which energy can be transferred from the knee to the ankle was defined as the knee-to-ankle joint energy transfer potential and the fraction of contact time during which energy can be transferred from the ankle to the knee was defined as the ankle-to-knee joint energy transfer potential^4^. The fraction of the contact time when the ankle and knee angles are in phase, i.e. the possibility of energy transfer from the ankle to the knee and vice versa, was defined as the energy transfer potential during the stance phase. To identify possible links between the energy transfer potential between the two joints and the management of total CoM energy, we calculated the changes in total CoM energy during the ankle-to-knee and knee-to-ankle energy transfer phases. Circular statistics^31,32^ were used to calculate the average coupling angle at each percentage of the respective stance phases.

We measured the EMG activity of the GM, GL, VM and VL muscles bilaterally with a wireless system (Myon m 320RX, Myon AG, Baar, Switzerland) at a sampling frequency of 2000 Hz. The EMG signals were first filtered with a fourth order high-pass zero-phase Butterworth filter with a cut-off frequency of 20 Hz, then rectified, and finally filtered with a low-pass zero-phase Butterworth filter with a cut-off frequency of 20 Hz. The filtered EMG data were normalized to the maximum EMG activity obtained during a maximal voluntary fixed-end plantarflexion contraction at an ankle angle of 0° for the GM and GL muscles and during a knee joint extension at 120° knee angle for the VM and VL muscles for each participant respectively. Using the first-order differential equation proposed by Zajac^33^, we estimated muscle activation as follows (Eq. 2):

2 $$\frac{d\widehat{\alpha}\left(t\right)}{dt}+\left[\frac{1}{{\tau}_{act}}\cdot\left(\beta+\left[1-\beta\right]\widehat{u}\left(t\right)\right)\right]\cdot\widehat{\alpha}\left(t\right)=\left(\frac{1}{{\tau}_{act}}\right)\cdot\widehat{u}\left(t\right)$$

Where $\widehat{\alpha}$ is the estimated muscle activation, $\widehat{u}$ is the measured normalized EMG-activity.

Based on the data from^34,35^, we assumed a 50% type I and 50% type II fiber distribution for the GM and GL muscles and a 38% type I and 62% type II distribution for VL and VM muscles to calculate the time activation constant (τ_act_) and the ratios of activation to deactivation time constant (β)^36^. An average weighted activation of the GM and GL as well as VM and VL muscles was calculated according to their volume ratios GM: 0.67, GL: 0.33^37^, and VM: 0.43, VL: 0.57^14^, respectively in order to achieve representative activation of the gastrocnemii and vasti muscles.

### Statistics

A linear mixed model was used to test for a main effect of locomotor condition (level walking vs. hole negotiation) on the outcomes investigated, i.e. temporal and spatial parameters, joint kinematics, CoM energy, muscle activation, energy transfer potential. Participants were considered as a random effect in the statistics, while locomotor condition was considered as a fixed effect. The model included only one random intercept per participant. In case of main effects, a post hoc analysis was conducted using the control for false discovery rate suggested by Benjamini and Hochberg^38^ and adjusted p-values are reported. A linear mixed model was also used to examine the association between energy transfer potential and changes in the total CoM energy during the energy transfer phases of the preparation, hole and recovery steps. The statistical analyses were performed using R version 4.0.1 (R Foundation for Statistical Computing, Vienna, Austria), and the ’nlme’ and ’emmeans’ packages were used for the linear mixed model and post hoc testing respectively. The significance level was set at α = 0.05 for all test comparisons. We also calculated Cohen’s effect size (d), with values of d < 0.2 indicating small effects, 0.2 ≤ d < 0.8 indicating medium effects, and d ≥ 0.8 indicating large effects^39^.

## Results

**Fig. 2:**
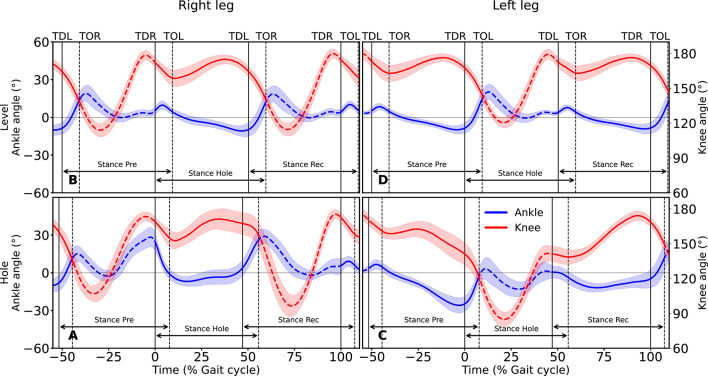
Ankle and knee joint angles of the right **(A**,** B)** and left **(C**,** D)** leg during level and hole negotiation walking. Positive values for the ankle angle represent a plantarflexed joint position and negative values represent a dorsiflexed joint position (note that the ankle joint angle is presented on the left vertical axis and the knee joint angle on the right vertical axis). The curves and shaded areas represent mean ±standard deviation. The solid part of the curves indicates stance phase and the dashed part indicates swing phase (note that the right leg has contact with the ground during the hole step and the left leg during the preparation and recovery steps). The horizontal axis is normalized to the gait cycle before touch-down in the hole (negative percentages) and to the gait cycle after touch-down in the hole (positive percentages). The vertical solid lines show the touch-down of the left and the right leg and the vertical dashed lines show the take-off. TDL: touch-down of the left leg, TOR: take-off of the right leg, TDR: touch-down of the right leg, TOL: take-off of the left leg. Stance Pre:stance phase of the preparation step, Stance Hole: stance phase of the hole step, Stance Rec: stance phase of the recovery step.

In the three steps examined, the total CoM energy only at touch-down of the preparation (*p* = 0.321, d = 0.14) and at take-off of the recovery (*p* = 0.871, d = 0.01) steps did not differ between the two gait conditions (Fig. 1; Table 1). In all other cases, the total CoM energy was significantly lower in the hole negotiation gait (preparation step: *p* < 0.001, d = 2.57 for take-off; hole step: *p* < 0.001, d = 1.34 for touch-down and *p* < 0.001, d = 1.72 for take-off; recovery step: *p* < 0.001, d = 1.70 for touch-down) (Table 1). Stance duration (*p* < 0.001, d = 0.91) and step length (*p* < 0.001, d = 0.85) in the recovery step, as well as step length (*p* < 0.001, d = 0.91) in the hole step, were greater during the hole negotiation gait (Table 1). No significant differences were observed in average horizontal CoM velocity between the two walking conditions during the preparation, hole and recovery steps (*p* = 0.725, d = 0.04; *p* = 0.623, d = 0.08; *p* = 0.799, d = 0.04, respectively) (Table 1). A greater range of dorsiflexion at the ankle joint was observed during the stance phase of both the preparation (*p* < 0.001, d = 3.25) and hole (*p* < 0.001, d = 3.87) steps in the hole negotiation gait (Fig. 2; Table 1). A significantly greater range of plantarflexion occurred at the ankle joint during the stance phase of the hole (*p* < 0.001, d = 2.31) and recovery (*p* = 0.001, d = 0.76) steps in hole negotiation (Fig. 2; Table 1). A greater range of flexion was observed at the knee joint during the stance phase of the preparation step (*p* < 0.001, d = 2.73), whereas lower flexion was observed during the hole step (*p* < 0.001, d = 2.51) (Fig. 2; Table 1). During the stance phase of the recovery step, the knee joint angle increased (Fig. 2; Table 1).

**Table 1:** Total centre of mass (CoM) energy at touch-down (TD) and take-off (TO), duration of the stance phase (stance time), step length, average horizontal CoM velocity (V_x_CoM_average_), range of ankle dorsiflexion, range of ankle plantarflexion, and range of knee flexion during the stance phase of the preparation, hole and recovery steps in level and hole negotiation gait (mean ± standard deviation). Note that the joint angles of the left leg are presented in the preparation and recovery steps, and the joint angles of the right leg are presented in the hole step. *: Statistically significant difference to level walking (*p* < 0.05).

**Variables**	**Preparation step**			**Step in the hole**		**Recovery step**	
	**Level walking**		**Hole negotiation**	**Level walking**	**Hole negotiation**	**Level walking**	**Hole negotiation**
CoM_energy_ at TD (J/kg)	10.71 ±0.52		10.78 ±0.52	10.69 ±0.49	10.01 ±0.52 *	10.65 ±0.48	9.74 ±0.59 *
CoM_energy_ at TO (J/kg)	10.87 ±0.50		9.69 ±0.44 *	10.80 ±0.48	10.05 ±0.54 *	10.82 ±0.47	10.82 ±0.44
Stance time (ms)	632 ±45		650 ±58	642 ±47	622 ±42	636 ±47	674 ±57 *
Step length (cm)	71.3 ±4.9		70.3 ±6.5	67.7 ±5.1	73.9 ±8.4 *	70.6 ±5.0	75.0 ±5.2 *
V_x_CoM_average_ (m/s)	1.32 ±0.12		1.33 ±0.13	1.32 ±0.13	1.32 ±0.14	1.31 ±0.11	1.32 ±0.13
Ankle dorsiflexion (°)	15.2 ±2.6		29.9 ±6.3 *	16.6 ±3.3	32.9 ±5.0 *	14.0 ±2.7	14.1 ± 5.4
Ankle plantarflexion (°)	25.3 ±4.0		25.5 ±7.2	26.1 ±3.1	35.5 ±4.9 *	24.3 ±5.1	29.2 ±8.1 *
Range knee flexion (°)	27.8 ±4.9		47.8 ±9.2 *	28.0 ±5.1	12.1 ±8.7 *	26.4 ±7.1	5.4 ±7.8 *

**Fig. 3:**
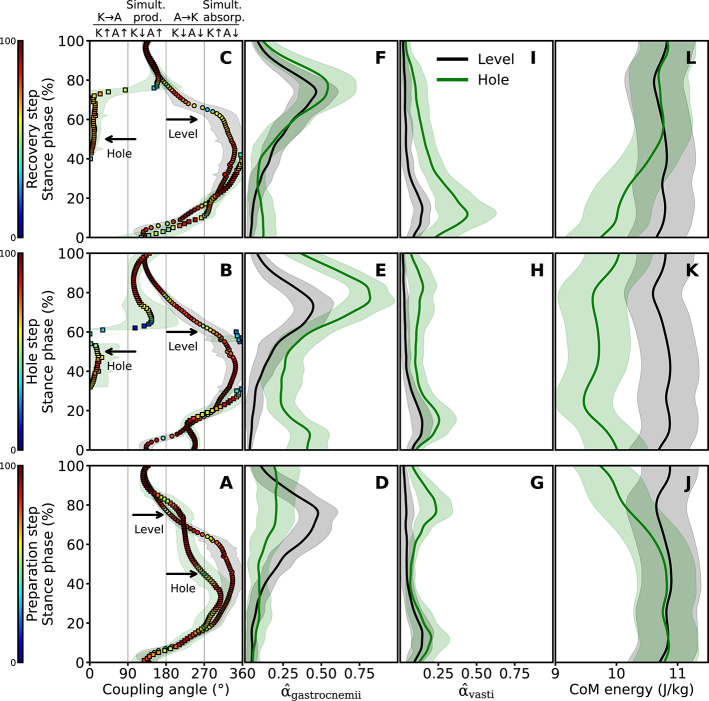
Coupling angles of the ankle and knee joint angles **(A**,** B**,**C)**, weighted activation of the gastrocnemii muscles ($\widehat{\alpha}$_gastrocnemii_) **(D**,** E**,**F)**, weighted activation of the vasti muscles ($\widehat{\alpha}$_vasti_) **(G**,** H**,**I)** and total centre of mass (CoM) energy **(J**,** K**,**L)** in the time-normalized stance phase (vertical axis) of the preparation, hole and recovery steps during level and hole negotiation walking. 0° < coupling angle ≤ 90^o^ indicates in-phase fluctuations with increasing of knee (K↑) and ankle (A↑) joint angles and potential for knee-to-ankle energy transfer (K→A) via the biarticular gastrocnemii muscles, 90° < coupling angle ≤ 180° indicates anti-phase fluctuations with decreasing of knee (K↓) and increasing ankle (A↑) joint angles and potential for simultaneous energy production (Simult. prod.) at the ankle and knee joint via the biarticular gastrocnemii muscles, 180° < coupling angle ≤ 270° indicates in-phase fluctuations with decreasing of knee (K↓) and ankle (A↓) joint angles and potential for ankle-to-knee joint energy transfer (A→K) via the biarticular gastrocnemii muscles, 270° < coupling angle ≤ 360° indicates anti-phase fluctuations with increasing of knee (K↑) and decreasing ankle (A↓) joint angles and potential for simultaneous energy absorption (Simult. absorb.) at the ankle and knee joint via the biarticular gastrocnemii muscles. The colour scale in the coupling angles represents the relative frequency of the participants in each of the four phases for every percentage of the stance phase. The curves and shaded areas represent the mean ± variability for the coupling angles and the mean ± standard deviation for all other variables.

There was a shift from simultaneous energy absorption via the biarticular gastrocnemii muscles to ankle-to-knee joint energy transfer during the preparation step and to knee-to-ankle joint energy transfer during the hole and recovery steps in hole negotiation compared to level walking (Fig. 3A, B,C). The maximum weighted activation of the gastrocnemii muscles was significantly lower during the stance phase of the preparation step (*p* < 0.001, d = 1.30), but significantly higher during the hole (*p* < 0.001, d = 3.58) and recovery (*p* = 0.002, d = 0.62) steps in hole negotiation gait (Fig. 3D, E,F; Table 2). The maximum weighted activation of the vasti muscles was significantly higher in all three steps of the hole negotiation gait (preparation: *p* < 0.001, d = 1.58; hole: *p* < 0.001, d = 1.72; recovery: *p* < 0.001, d = 2.42) (Fig. 3G, H,I; Table 2). The peak-to-peak range of total CoM energy during the stance phase was significantly greater during hole negotiation than during level walking for the preparation (*p* < 0.001, d = 5.50), hole (*p* < 0.001, d = 2.81), and recovery (*p* < 0.001, d = 5.49) steps (Fig. 3J, K,L; Table 2).

**Table 2:** Peak-to-peak range of total centre of mass energy (CoM_energy, range_), maximum weighted activation of the gastrocnemii muscles ($\widehat{\alpha}$_max gastrocnemii_) and maximum weighted activation of the vasti muscles ($\widehat{\alpha}$_max vasti_) during the stance phase of the preparation, hole and recovery steps in level and hole negotiation gait (mean ± standard deviation). Note that the muscle activations of the left leg are presented in the preparation and recovery steps, and the muscle activations of the right leg are presented in the hole step. *: Statistically significant difference to level walking (*p* < 0.05).

**Variables**	**Preparation step**			**Step in the hole**		**Recovery step**	
	**Level walking**	**Hole negotiation**		**Level walking**	**Hole negotiation**	**Level walking**	**Hole negotiation**
CoM_energy, range_ (J/kg)	0.30 ±0.05	1.21 ±0.23 *		0.33 ±0.06	0.66 ±0.15 *	0.29 ±0.08	1.67 ±0.21 *
$\widehat{\alpha}$_max gastrocnemii_	0.21 ±0.06	0.13 ±0.07 *		0.18 ±0.06	0.44 ±0.08 *	0.20 ±0.05	0.24 ±0.08 *
$\widehat{\alpha}$_max vasti_	0.06 ±0.04	0.14 ±0.05 *		0.06 ±0.03	0.14 ±0.06 *	0.06 ±0.04	0.19 ±0.07 *

**Fig. 4:**
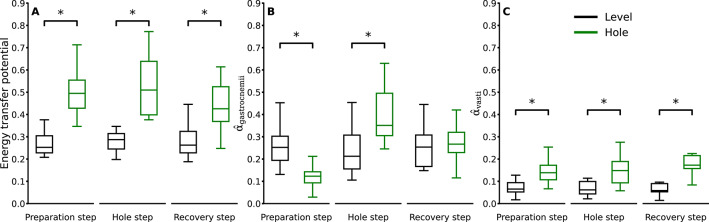
Energy transfer potential via the biarticular gastrocnemii muscles **(A)**, weighted activation of the gastrocnemii ($\widehat{\alpha}$_gastrocnemii_) **(B)** and vasti muscles ($\widehat{\alpha}$_vasti_) **(C)** in the energy transfer phases during the stance phase of the preparation, hole, and recovery steps while level walking and hole negotiation. * Statistically significant (*p* < 0.05) differences.

We found a significant increase in energy transfer potential between the knee and ankle joints during the stance phase of the preparation (*p* < 0.001, d = 2.08), hole (*p* < 0.001, d = 2.23) and recovery (*p* = 0.008, d = 1.0) steps in the hole negotiation compared to level walking (Fig. 4A). During the energy transfer phases, the activation of the gastrocnemii muscles was significantly lower during the preparation step (*p* < 0.001, d = 1.62) and significantly higher during the hole step (*p* < 0.001, d = 1.42) in hole negotiation than in level walking (Fig. 4B). In the recovery step, there were no significant differences in the activation of the gastrocnemii during the energy transfer phases between the two conditions (*p* = 0.182, d = 0.27) (Fig. 4B). The weighted activation of the vasti muscles was significantly greater during the energy transfer phases in all three steps (preparation: *p* < 0.001, d = 1.20; hole: *p* < 0.001, d = 1.36; recovery: *p* < 0.001, d = 2.31) in hole negotiation (Fig. 4C).

**Fig. 5:**
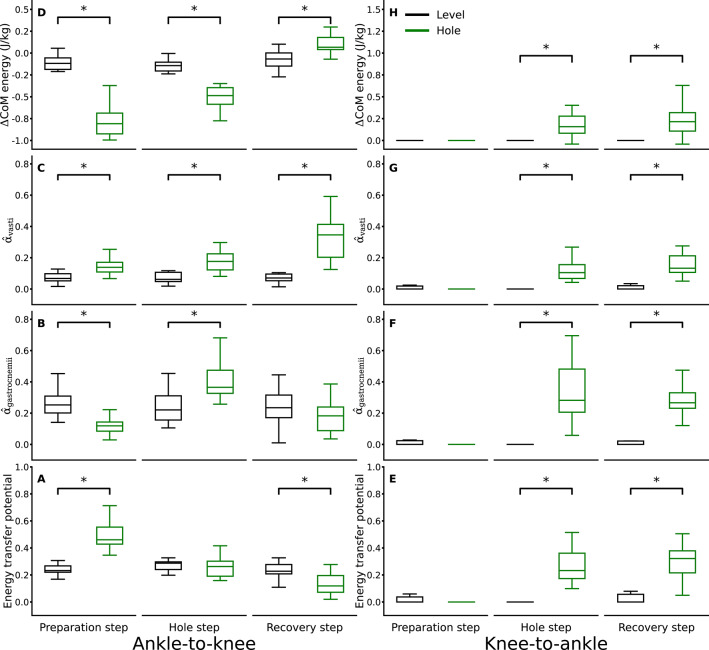
Ankle-to-knee joint **(A)** and knee-to-ankle joint **(E)** energy transfer potential via the biarticular gastrocnemii muscles, weighted activation of the gastrocnemii muscles ($\widehat{\alpha}$_gastrocnemii_) **(B**,** F)**, weighted activation of the vasti muscles ($\widehat{\alpha}$_vasti_) **(C**,** G)**, and change in total center of mass energy (ΔCoM energy) **(D**,** H)** in the ankle-to-knee and knee-to-ankle joint energy transfer phases during the stance phase of the preparation, hole, and recovery steps while level walking and hole negotiation. * Statistically significant (*p* < 0.05) differences.

During the preparation step, the energy transfer potential from the ankle to the knee joint was significantly higher (*p* < 0.001, d = 2.34) in the hole negotiation gait, whereas the energy transfer potential from the knee to the ankle joint was close to zero in both gait conditions and did not differ (*p* = 0.281, d = 0.35) between them (Fig. 5A, E). There was no significant difference in the ankle-to-knee joint energy transfer potential (*p* = 0.486, d = 0.24) between level walking and hole negotiation during the hole step (Fig. 5A). The ankle-to-knee joint energy transfer potential was significantly lower in the recovery step (*p* < 0.001, d = 1.28) during hole negotiation (Fig. 5A). The knee-to-ankle joint energy transfer potential increased significantly during the hole (*p* < 0.001, d = 2.65) and recovery (*p* < 0.001, d = 2.14) steps in the hole negotiation gait (Fig. 5E). The average weighted activation of the gastrocnemii muscles during the ankle-to-knee joint energy transfer phase was lower (*p* < 0.001, d = 1.71) in the preparation step and higher (*p* < 0.001, d = 1.63) in the hole step of the hole negotiation gait (Fig. 5B). In both the hole and recovery steps, the average weighted activation of the gastrocnemii muscles during the knee-to-ankle joint energy transfer phase was significantly higher (*p* < 0.001, d = 2.25 and *p* < 0.001, d = 2.06) in hole negotiation compared to level walking (Fig. 5F). The average weighted activation of the vasti muscles during the ankle-to-knee joint energy transfer phase of the preparation (*p* < 0.001, d = 1.12), hole (*p* < 0.001, d = 1.74), and recovery (*p* < 0.001, d = 2.64) steps was significantly greater in hole negotiation (Fig. 5C). While negotiating the hole, the average weighted activation of the vasti was also significantly greater during the knee-to-ankle joint energy transfer phase of the hole (*p* < 0.001, d = 2.47) and recovery (*p* < 0.001, d = 2.57) steps (Fig. 5G). We found a greater decrease in CoM energy during the ankle-to-knee joint energy transfer phase in both the preparation and hole steps of the hole negotiation gait (*p* < 0.001, d = 4.52 and d = 2.51, respectively), whereas an increase in CoM energy was observed in the recovery step (Fig. 5D). We also found a higher increase in CoM energy during the knee-to-ankle joint energy transfer phase in the hole (*p* = 0.002, d = 1.73) and recovery (*p* < 0.001, d = 1.72) steps in the hole negotiation gait (Fig. 5H).

**Fig. 6:**
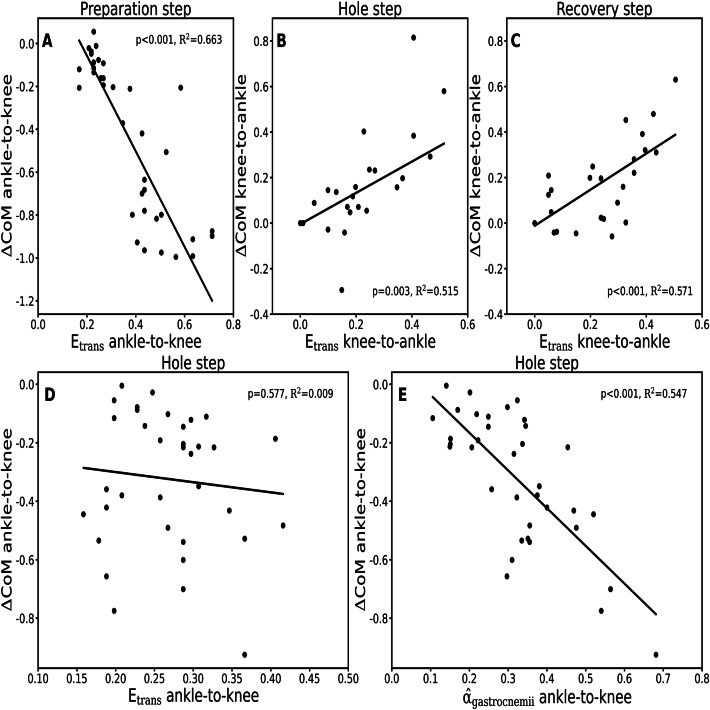
Relationship between ankle-to-knee joint energy transfer potential (E_trans_ ankle-to-knee) and changes in total centre of mass energy in the ankle-to-knee joint energy transfer phase (ΔCoM ankle-to-knee) during stance of the preparation step **(A)**, relationships between knee-to-ankle joint energy transfer potential (E_trans_ knee-to-ankle) and changes in ΔCoM in the knee-to-ankle joint energy transfer phase (ΔCoM knee-to-ankle) during stance of the hole **(B)** and recovery **(C)** steps, relationship between E_trans_ ankle-to-knee and ΔCoM ankle-to-knee during stance of the hole step **(D)**, and relationship between weighted activation of the gastrocnemii muscles in the ankle-to-knee joint energy transfer phase ($\widehat{\alpha}$_gastrocnemii_ ankle-to-knee) and ΔCoM ankle-to-knee during stance of the hole step **(E)**.

There was a significant (*p* < 0.001) relationship between ankle-to-knee joint energy transfer potential and the reduction in CoM energy during the ankle-to-knee joint energy transfer phase of the preparation step (Fig. 6A). A significant relationship was also found between the knee-to-ankle joint energy transfer potential and the increase in total CoM energy during the knee-to-ankle joint energy transfer phases of the hole (*p* = 0.003) and recovery (*p* < 0.001) steps (Fig. 6B, C). Although there was no significant relationship between the ankle-to-knee joint energy transfer potential and the change in total CoM energy in the hole step (*p* = 0.577), the weighted activation of the gastrocnemii muscles was significantly (*p* < 0.001) related to the change in total CoM energy during the ankle-to-knee joint energy transfer phase (Fig. 6D, E).

## Discussion

The findings demonstrate the involvement of biarticular mechanisms of the gastrocnemii muscles during walking on uneven terrain, such as negotiating a hole. Furthermore, the found decrease in total CoM energy during the ankle-to-knee joint energy transfer phase in the preparation and hole steps, and increase during the knee-to-ankle joint energy transfer phase in the hole and recovery steps, indicate an important contribution of the gastrocnemii muscles’ biarticular mechanisms in controlling the increased challenge of managing total CoM energy during hole negotiation gait.

During the hole negotiation gait, the total CoM energy began to decrease from the midpoint of the stance phase of the preparation step and continued until approximately 25% of the stance phase of the hole step. The energy reduction was 1.34 ± 0.41 J/kg, which is equivalent to 12% of the total CoM energy during level walking, indicating a substantial amount of energy absorbed by the musculoskeletal system during this period. Subsequently, the total CoM energy increased until the midpoint of the stance phase of the recovery step, indicating energy production by the musculoskeletal system. The greater range of ankle dorsiflexion and knee flexion of the stance leg in the preparation step may contribute to CoM energy absorption due to the body lowering strategy typically used during hole negotiation^22,24,40^. The greater ankle plantarflexion in the first part of the stance phase in the hole step is again an important stabilising strategy that contributes to the necessary absorption of the body’s kinetic energy^22,24^. Similarly, the earlier and greater plantarflexion during the stance phase of the hole and recovery steps and the greater knee extension during the stance phase of the recovery step may contribute to the increased total CoM energy until the mid-stance phase of the recovery step. Nevertheless, the observed increase in ankle-to-knee joint energy transfer potential during the stance phase of the preparation step, as well as the increased knee-to-ankle joint energy transfer potential during the stance phase of the hole and recovery steps, indicate an important participation of biarticular mechanisms of the gastrocnemii muscles to the required management of total CoM energy during the negotiation of the hole.

A unique feature of the structure and function of biarticular muscles is their ability to generate moments at two joints simultaneously. This allows them to actively transfer mechanical power and energy between the two spanning joints^2,8^. During level walking, humans exhibit inverted pendulum dynamics with an exchange between the potential and kinetic energy of the CoM^41,42^, resulting in minimal changes in total CoM energy. During hole-negotiation gait, the peak-to-peak range of the total CoM energy during the stance phase of the preparation, hole, and recovery steps increased significantly, by a factor of between 2.0 and 5.6, reflecting an increased demand to manage total CoM energy. The increase in energy transfer potential during the hole negotiation compared to level walking was 82% in the preparation step, 79% in the hole step and 40% in the recovery step. During the ankle-to-knee joint energy transfer phase, the weighted activation of the gastrocnemii muscles increased 1.7-fold in the hole step. During the knee-to-ankle joint energy transfer phase, it increased 11-fold in the hole step and 5.6-fold in the recovery step. Although the gastrocnemii muscles had 49% lower weighted activation during the ankle-to-knee joint energy transfer phase of the preparation step in hole negotiation gait than during level walking, they remained active and increased their activation patterns during this phase. Taken together, these results show that during the specific phases of stance where an energy transfer from the ankle to the knee joint and vice versa is possible, the activated gastrocnemii muscles could generate active forces and perform mechanical power at the ankle and knee joints, i.e. conditions for transfer of energy by biarticular mechanisms during the negotiation of the hole. Furthermore, we found significant associations between the ankle-to-knee joint energy transfer potential and the decreased total CoM energy during the energy transfer phase from the ankle to the knee in the preparation step. We also found significant associations between the knee-to-ankle joint energy transfer potential and the increased total CoM energy during the energy transfer phase from the knee to the ankle in the hole and recovery steps. Finally, we observed a significant correlation between the weighted activation of the gastrocnemii muscles and the reduction in total CoM energy during the ankle-to-knee joint energy transfer phase of the hole step. These associations support the argument that the biarticular mechanisms of the gastrocnemii muscles play a significant role in managing total CoM energy during hole negotiation, which is a more challenging locomotor task in terms of controlling total CoM energy than level walking.

At the beginning of the stance phase of the hole step and during the ankle-to-knee joint energy transfer phase, the mean weighted activation of the gastrocnemii muscles was 41% of the maximum, which indicates active moment generation at the ankle and knee joints by the gastrocnemii muscles. At the knee joint, the gastrocnemii muscles generate flexion moments that increase the active extension moments generated by the knee extensor muscles, particularly the vasti muscles, which are approximately 5.7 times larger than the rectus femoris muscle^43^. The mean weighted activation of the vasti muscles during the ankle-to-knee joint energy transfer phase was 18% of the maximum, indicating that the vasti were active and generated forces. At the same time, the knee joint is flexing, i.e., the vasti muscle-tendon units are lengthened, so that most of the energy transferred from the ankle to the knee joint via the gastrocnemii muscles will be absorbed by the muscle-tendon units of the vasti muscles. Irrespective of whether the contractile elements of the vasti absorbed energy during this phase, part of the transferred energy will be stored as elastic energy in their tendinous structures. During the subsequent phase of energy transfer from the knee to the ankle joint, the mean weighted activation of the gastrocnemii muscles was 34% of the maximum, whereas that of the vastus muscles was 12%. As the knee joint extended during this phase, the vasti muscle-tendon units actively shortened. The active shortening of the vasti muscle-tendon units indicates a transfer of energy to the ankle joint via the gastrocnemii muscles from the vasti muscles. Some of this energy could originate from the stored elastic energy previously transferred from the ankle to the knee joint by the gastrocnemii muscles. It appears that the contribution of the biarticular gastrocnemii to the management of total CoM energy during hole negotiation is accompanied by an exchange of energy between elastic tissues, as has been reported during jumping and running^2,17^. Furthermore, the increased weighted activation of the vasti muscles during the ankle-to-knee joint energy transfer phase of the preparation step and during the knee-to-ankle joint energy transfer phase of the recovery step implies that the monoarticular vasti are also involved in the absorption and production of total CoM energy via biarticular mechanisms in these steps. The active lengthening of the vasti muscle-tendon units during the energy transfer phase from the ankle to the knee joint in the preparation step indicates that the vasti muscles are absorbing energy, some of which is transferred to the knee joint via biarticular mechanisms of the gastrocnemii muscles. Conversely, the active shortening of the vasti muscle-tendon units during the energy transfer phase from the knee to the ankle joint in the recovery step indicates that the vasti muscles are producing energy, some of which is transferred via the gastrocnemii biarticular mechanisms to the ankle joint.

It should be noted that the reported potentials of energy transfer from the ankle to the knee joint and vice versa by the biarticular gastrocnemii muscles are metrics that quantify the fraction of contact time that energy transfer between the two joints is possible and not the amount of energy transferred. The quantification of the amount of energy transferred between the two joints requires the calculation of the gastrocnemii mechanical power at the ankle and knee joints^15–17^. For example, in inactive gastrocnemii muscles, the magnitude of energy transfer between the two joints is very low^16^. However, the mean weighted activation of the gastrocnemii muscles during the hole negotiation gait was between 13 and 41% of the maximum during the part of the contact time when energy could be transferred between the ankle and knee joints. This indicates that the gastrocnemii muscles generated moments and mechanical power at both the ankle and knee joints, thus transferring energy between the two joints. In the future, validated musculoskeletal models that can accurately predict muscle forces and experimental approaches that can quantify the energy transfer between joints via biarticular muscles during locomotion would improve our understanding of these mechanisms. In our experimental design, we used a fixed hole depth of 15 cm for the hole negotiation trials. In the real world, however, a variety of depth holes have to be managed, and the involvement of the gastrocnemii’s biarticular mechanisms may differ. Finally, it should be mentioned that the concept of energy transfer between lower leg joints via biarticular muscles extends beyond energy transfer between the ankle and knee via the gastrocnemii muscles. There are also possibilities for energy transfer from the hip to the knee, and vice versa, via the biarticular rectus femoris and hamstrings. Investigating these possibilities could be very important for improving our understanding of human locomotion in uneven terrain, and could be investigated in future studies using the approach presented here.

This study is the first to report a link between the potential for energy transfer between the ankle and knee joints via the biarticular gastrocnemii muscles and the changes in total CoM energy during hole negotiation gait a locomotor task that is more challenging than level walking in terms of managing total CoM energy. Furthermore, the increased potential for energy transfer between the ankle and knee joints through the biarticular gastrocnemii muscles, combined with their active state during the energy transfer phases, indicates a significant involvement of biarticular mechanisms during the hole negotiation gait. These insights are valuable for the development of prevention and rehabilitation treatments that aim to improve stability control in healthy and pathological individuals. For example, training exercises that trigger in-phase fluctuations between the ankle and knee joints could improve prevention and rehabilitation treatments by teaching individuals to utilise these mechanisms quickly. They can also be important for the development of prostheses and exoskeletons that support stable movement on uneven terrains, and for the control of bipedal robots.

## Data Availability

The original data of the study can be accessed here https://doi.org/10.6084/m9.figshare.29582981.v1.
